# Extra‐articular involvement of rheumatoid arthritis in three seropositive patients in the absence of initial joint involvement

**DOI:** 10.1002/iid3.514

**Published:** 2021-09-02

**Authors:** Razvan M. Chirila, Florentina Berianu, Andy Abril, Ronald R. Butendieck

**Affiliations:** ^1^ Division of General Internal Medicine Mayo Clinic Jacksonville Florida USA; ^2^ Division of Rheumatology Mayo Clinic Jacksonville Florida USA

**Keywords:** autoimmune diseases, human, rheumatoid arthritis

## Abstract

**Introduction:**

Extra‐articular involvement (EAI) in rheumatoid arthritis (RA) is rare, severe and usually presents after years of joint involvement. Onset of RA as lung involvement has been published. We describe a series of three patients with strongly positive anti‐citrullinated peptide antibodies (ACPA) and rheumatoid factor (RF) positive in the absence of joint symptoms, but with significant EAI.

**Methods:**

This is a descriptive case series of three patients evaluated in an academic medical center rheumatology clinic.

**Results:**

A 50‐year‐old female presented with severe recurrent scleritis in the background of strongly positive ACPA, but no joint involvement. Her ocular disease responded well to rituximab. Four years later, she developed peripheral inflammatory arthritis consistent with RA. A 74‐year‐old male presented after developing recurrent steroid‐dependent serositis (pleuro‐pericarditis) and interstitial lung disease (ILD). Serology was notable for strongly positive ACPA, but no joint involvement. Serositis responded well to adalimumab. Two years after initial symptoms, he developed peripheral joint involvement after holding adalimumab. A 56‐year‐old female presented with an isolated, biopsy‐proven subcutaneous rheumatoid nodule. Subsequently, she developed pancytopenia and fatigue. She tested strongly positive for ACPA and RF. Bone marrow biopsy was negative for malignancy and she had no evidence of infection. She responded to steroids and hydroxychloroquine and had not developed joint involvement after 2 years of follow‐up.

**Conclusion:**

EAI as an onset of RA is a complex and not easily recognized entity if typical joints involvement is not yet present. Early diagnosis may help guide specific therapy and prevent sequelae and co‐morbidities.

## 1 INTRODUCTION

Extra‐articular involvement (EAI) or systemic manifestations of rheumatoid arthritis (RA) include rheumatoid nodules, interstitial lung disease, vasculitis with potential cutaneous and neuropathic involvement, serositis and eye disease.^1^ The hallmark of RA is prolonged morning stiffness and symmetric inflammatory arthritis involving the small joints of the hands and feet. Isolated EAI of RA without joint manifestations is exceptionally rare, except for lung involvement.

Anti‐citrullinated peptide antibodies (ACPA) are present in the serum of 80%–90% of RA patients and are more specific for RA than rheumatoid factor (RF), with specificity approaching 90%.^2^ Like the RF, ACPA can appear long before the onset of clinical arthritis and could be a marker for immune hyperreactivity and subclinical inflammation.^3^ ACPA is a predictor of more aggressive disease and is accompanied by bone and cartilage destruction.^4^ A cohort of 74 patients with ACPA presenting with interstitial lung disease (ILD) consistent with established RA, but with no joint symptoms at the time of diagnosis, has been published. A few of these patients developed articular RA within a short period of follow‐up (2 years).^5^ We describe a series of three patients with ACPA and no initial joint involvement typical of RA, but with significant extra‐articular manifestations (Table 1).

**Table 1:** Clinical characteristics

Case	Serology	Morning stiffness	Arthritis	Constitutional symptoms	Serositis	Lung involvement	Eye	Rheumatoid nodules	ESR CRP at diagnosis	Other laboratory abnormalities
1	ACPA > 250; RF 31	None^a^	None^a^	None	None	None	Scleritis	None	ESR 43	Normal
									CRP 1.2	
2	ACPA > 250; RF 75	None^a^	None^a^	None	Recurrent pleuro‐pericarditis	ILD	None	None	ESR 30; CRP 75	Normal
3	ACPA > 250; RF 200	None	None	Fatigue	None	None	None	Yes	ESR 5	Pancytopenia
									CRP 4.3	

*Note*: ACPA values: strongly positive >60 units/ml; CRP normal values: 0.0–8.0 mg/L; ESR normal values: 0–22 mm/h; RF normal values: 0–14 IU/ml.

Abbreviations: ACPA, anti‐citrullinated peptide antibodies; CRP, C‐reactive peptide; ESR, erythrocyte sedimentation rate; ILD, interstitial lung disease; RF, rheumatoid factor.

^a^ Patient developed joint pain and stiffness years later.

### 1.1 Cases

#### 1.1.1 Patient 1

**Figure 1:**
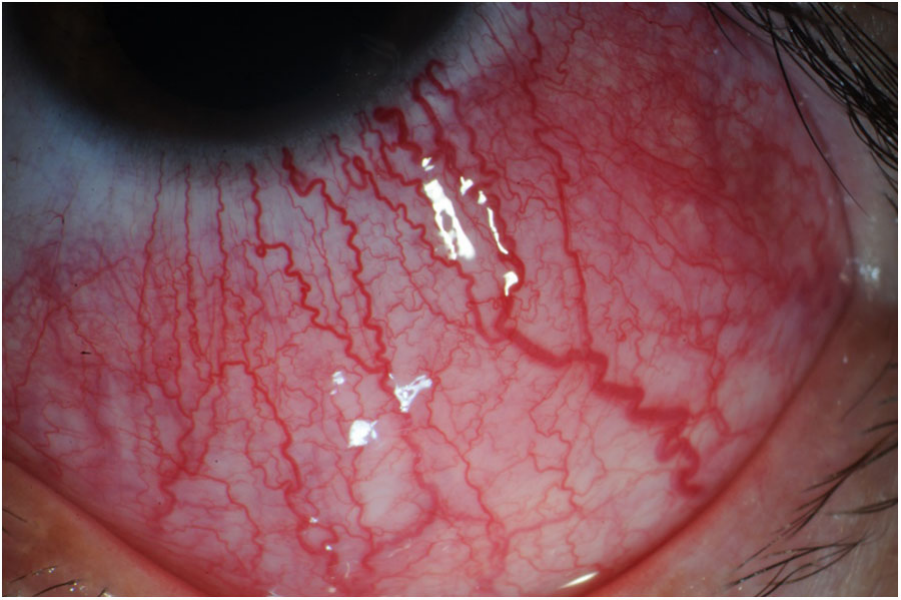
Typical image of scleritis

A 50‐year‐old African American female was followed in rheumatology clinic for 5 years. She developed severe, recurrent scleritis that involved either eye for 1 year before her presentation (Figure 1). Evaluation by ophthalmology did not identify any underlying infection through extensive testing for conditions like syphilis, herpes, Lyme disease, and tuberculosis. She was treated with topical glucocorticoids followed by high‐dose oral steroids to control active inflammation. Unfortunately, scleritis recurred with multiple attempts at glucocorticoid taper. She tested positive for RF (31 IU/ml with RF normal values: 0–14 IU/ml) and was strongly positive for ACPA (>250 units/ml with ACPA normal values: 0–19). Serologic testing for anti‐neutrophil cytoplasmic antibodies (ANCA), anti‐nuclear antibodies (ANA), extractable nuclear antibodies (ENA), and angiotensin‐converting enzyme returned negative/normal. Her chest X‐ray was unremarkable. Apart from scleritis, she had no other clinical manifestations, physical exam findings, or history to suggest systemic disease. She is a life‐long nonsmoker. There was no family history of rheumatoid arthritis.

Despite the lack of joint involvement at presentation, it was felt that the patient's scleritis was likely an extra‐articular manifestation of RA. She failed attempts at therapy with methotrexate and adalimumab. Secondary to the refractory nature of her disease, she was started on and had a remarkable clinical response to RA protocol rituximab (RTX) infusion therapy (two doses of 1 g each, separated by 2 weeks). She required repeated RTX infusions every 12 months for scleritis control. Four years after presentation to our institution, she developed episodes of inflammatory joint symptoms involving bilateral wrists and shoulders fulfilling the 2010 Rheumatoid arthritis classification criteria for RA.^6^ The first episode occurred 9 months after the last RTX infusion. After increasing RTX frequency to every 6–8 months, all inflammatory symptoms were controlled.

#### 1.1.2 Patient 2

**Figure 2:**
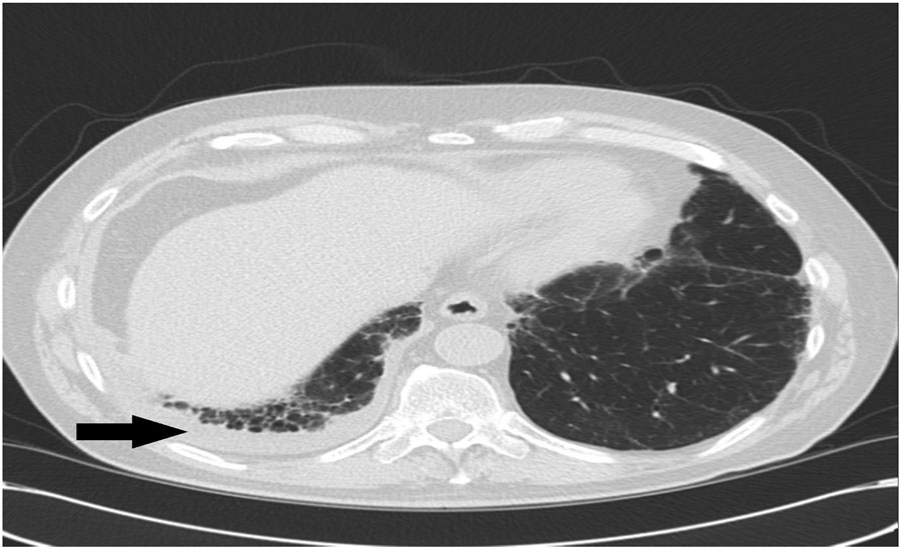
High resolution CT chest with small right pleural effusion (Black arrow). CT, computed tomography

A 74‐year‐old white male patient was followed in rheumatology clinic for 2 years. Six months before presentation, he had a mild episode of pericarditis treated with high doses of nonsteroidal anti‐inflammatory drugs for 6 weeks. He then experienced recurrent episodes of pleuritis that were documented on imaging, involving either lung. On one occasion, 200 ml of exudative pleural fluid was removed. Fluid analysis did not reveal findings suggestive of infection or malignancy. High‐resolution CT scan of the chest described a small pleural effusion (Figure 2) and scattered peripheral fibrosis with honeycombing, reticulation and traction bronchiectasis, suggestive of a usual interstitial pneumonitis pattern. Pulmonary function tests (PFTs) revealed reduced diffusion capacity for carbon monoxide and functional vital capacity at 64% of predicted. Repeat echocardiogram was negative for pericardial effusion and overall cardiac function was within the normal limits. Autoimmune work‐up revealed elevated RF (75 IU/ml) and strongly positive ACPA (>250 units/ml). Addition testing noted negative ANA, ENA panel, ANCA and normal C3 and C4 levels. Erythrocyte sedimentation rate (ESR) and C‐reactive protein (CRP) were elevated at 30 mm/h and 75 mg/L, respectively. He had no joint involvement or other constitutional symptoms. He is a life‐long nonsmoker. There was no family history of rheumatoid arthritis.

The episodes of serositis increased in frequency and required repeated steroid therapy. The patient was started on azathioprine but continued to have episodes of serositis. Treatment with adalimumab was initiated with resolution of serositis and normalization of inflammatory markers. After 6 months of therapy, adalimumab was stopped at the patient's request. Two months after discontinuation of adalimumab, he developed inflammatory joint symptoms involving bilateral 2–5th metacarpophalangeal joints, bilateral third proximal interphalangeal joints and bilateral wrists fulfilling the 2010 Rheumatoid arthritis classification criteria for RA.^6^ Joint involvement responded to reinitiation of adalimumab.

#### 1.1.3 Patient 3

A 56‐year‐old white female patient was followed in rheumatology clinic for 2 years. Four years before presentation, she was found to have a painful mass on the plantar aspect of her right foot. Pathology from surgical excision described subcutaneous necrobiosis and fibrinoid degeneration with palisaded histiocytic inflammation, compatible with a rheumatoid nodule. Apart from the rheumatoid nodule, patient was asymptomatic. She tested strongly positive for ACPA (>250 units/ml). RF was highly positive. ANA, ENA panel, C3, and C4 were negative/normal. In the subsequent months, she developed extreme fatigue and work up revealed a white blood cell count (WBC) of 1700/mm^3^ (normal: 3500–10,000) with a neutrophil count of 800/mm^3^ (normal: 1700–3500) and hemoglobin (Hgb) of 11.5 g/dl (normal: 12.0–15.5). There was no family history of rheumatoid arthritis. She was referred to this institution to rule out Felty syndrome. She had no recurrent fevers or signs of infection. She had no joint pain or stiffness. Physical exam was unremarkable for joint synovitis, splenomegaly, or rheumatoid nodules. No hepatosplenomegaly was found on abdominal ultrasound. Hematologic evaluation with bone marrow biopsy was remarkable for hypercellular bone marrow, increased myelopoiesis and lymphocytosis. Flow cytometry and immune phenotyping were negative for hematologic malignancies. It was felt that the reactive bone marrow process was in the context of anemia of chronic disease. Serum protein electrophoresis demonstrated polyclonal hyper‐gammaglobulinemia without monoclonal protein by immunofixation. She did not meet criteria for Felty syndrome, as she had no active joint involvement or splenomegaly. It was felt that her fatigue and hematologic abnormalities were likely extra‐articular manifestations of RA. She reported significant improvement of fatigue with a trial of prednisone 20 mg daily. After 1 month of prednisone therapy, blood counts improved (WBC 2200/mm^3^, neutrophils 1100/mm^3^) and Hgb remained stable. Several steroid‐sparing agents were attempted. Sulfasalazine was not tolerated. Methotrexate was suggested; however, the patient was reluctant to try this medication. She was initiated on hydroxychloroquine and low‐dose prednisone, which helped her fatigue and maintained cell counts. In the 2 years of follow‐up, she did not develop any arthritic symptomatology or new rheumatoid nodules.

## 2 DISCUSSION

EAI of RA occurs in up to 40% of patients over the lifetime of the disease.^4^, ^7^ Extra‐articular features vary with the duration and disease severity and are associated with excess mortality.^8^ Early diagnosis of these patients would help guide therapy and improve comorbidities and mortality.

We found ACPA and RF positivity in the patients described, who presented without joint involvement, but had typical extra‐articular manifestations of RA (Table 1). ACPA is reported to be highly specific for RA.^1^, ^2^ Multiple studies have shown that RA‐specific antibodies have been present in serum years before articular manifestations of RA.^2^ Our patients exhibited only EAI at the onset. Long‐term follow‐up may indicate that these patients eventually develop arthritis. Early diagnosis and initiation of appropriate therapy may prove to be crucial to prevent organ damage and sequelae from untreated local or systemic inflammation. In the cohort of 74 patients described by Fisher et al., three patients developed articular symptoms at 2 years of follow‐up.^5^ In our series, patient 1 developed RA symptoms four years after the diagnosis of scleritis and patient 2 developed RA symptoms 2 years after developing serositis. Rheumatoid vasculitis, Felty syndrome, serositis, RA pachymeningitis are rare manifestations that are associated with RF positivity. These usually occur in long‐standing RA, yet case reports of rheumatoid vasculitis or RA pachymeningitis as an onset, preceding joints involvement, have scarcely been described.^8^, ^9^, ^10^, ^11^, ^12^ We would like to raise awareness for clinicians to consider the possibility of EAI of RA in the absence of typical joint involvement, as checking specific serology will help with diagnosis and appropriate therapy selection.

## CONFLICT OF INTERESTS

The authors declare that there are no conflict of interests.
